# Supplementary material for: Dissecting ribosomal particles throughout the kingdoms of life using advanced hybrid mass spectrometry methods
Source: Nat Commun. 2018 Jun 27;9:2493. doi: 10.1038/s41467-018-04853-x (PMC6021402; doi:10.1038/s41467-018-04853-x)
Supplement: Supplementary file 1 — Supplementary Information [file 41467_2018_4853_MOESM1_ESM.pdf]

Supporting Information

Dissecting ribosomal particles throughout the kingdoms of life using advanced hybrid mass spectrometry methods

Michiel van de Waterbeemd[1,2,#], Sem Tamara[1,2,#], Kyle L Fort[1,2,3], Eugen Damoc[3], Vojtech Franc[1,2], Philipp Bieri[4], Martin Itten[4], Alexander Makarov[1,3], Nenad Ban[4], and Albert JR Heck[1,2,*]

1) Biomolecular Mass Spectrometry and Proteomics, Bijvoet Center for Biomolecular Research and Utrecht Institute for Pharmaceutical Sciences, Utrecht University, Utrecht 3584CH, the Netherlands
2) Netherlands Proteomics Center, Utrecht 3584CH, the Netherlands.
3) Thermo Fisher Scientific, 28199 Bremen, Germany
4) Department of Biology, Institute of Molecular Biology and Biophysics, ETH Zurich, 8093 Zurich, Switzerland

# These authors contributed equally

* Correspondence and requests for materials should be addressed to A.J.R.H. (email: a.j.r.heck@uu.nl)

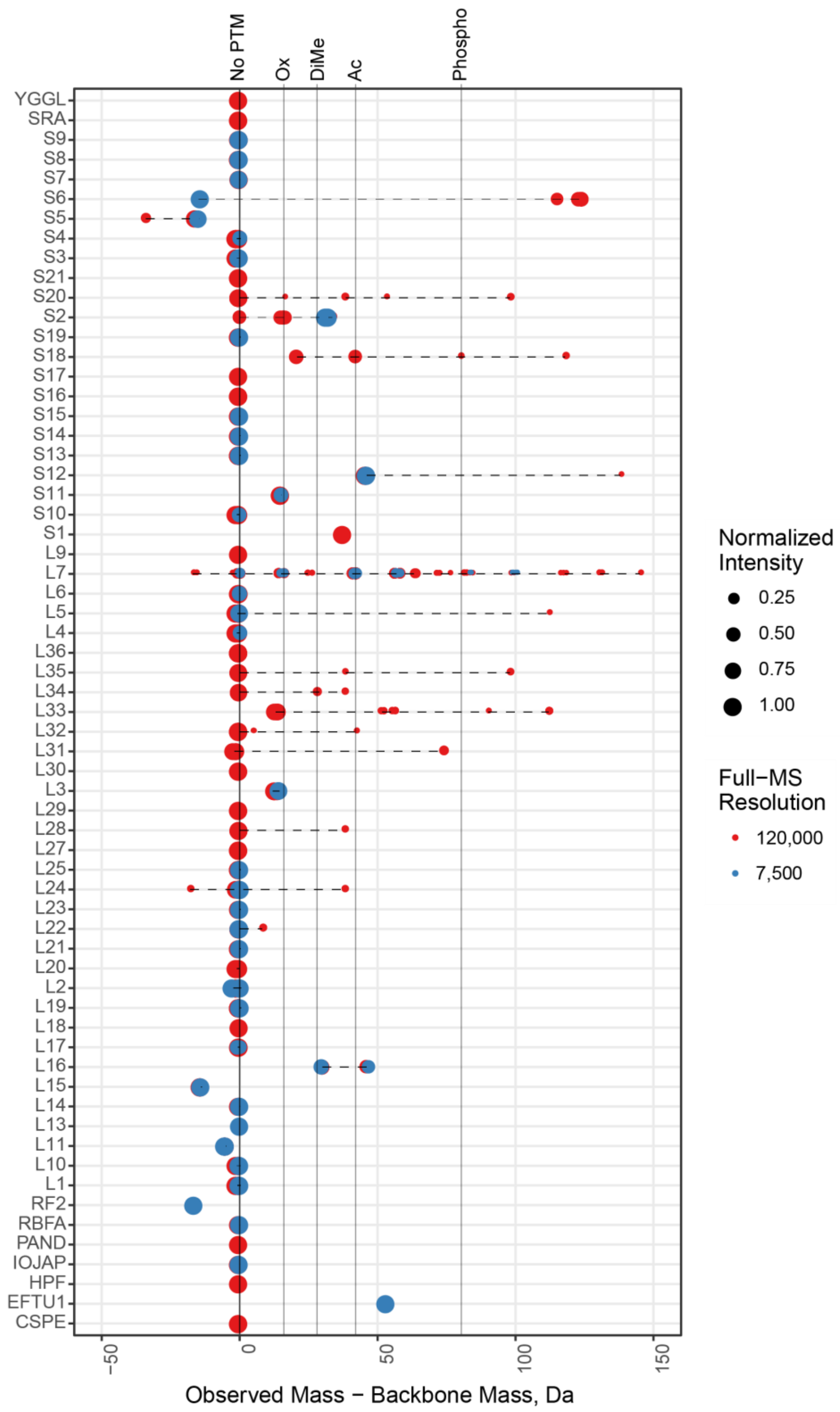

Supplementary Figure 1. Overview of post-translational modifications observed in the top-down analysis of *E. coli* ribosomal particles. The abundances of different proteoforms of ribosomal and non-ribosomal proteins detected by top-down LC-MS/MS in the Ec70S sample are indicated by sized filled circles.

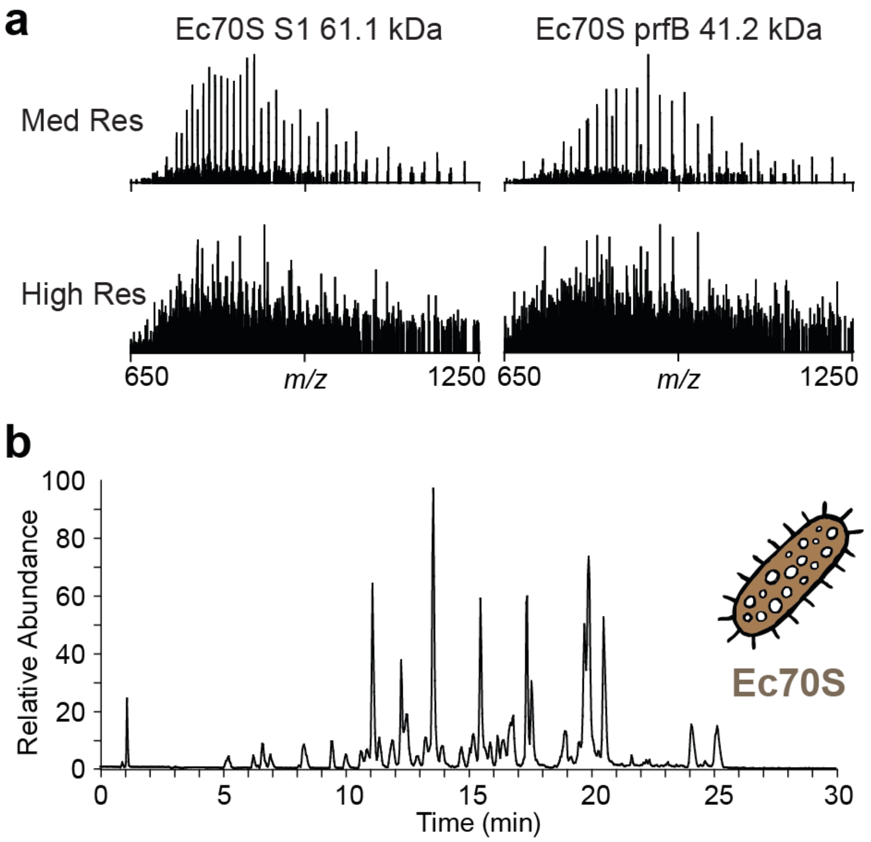

Supplementary Figure 2. Performance comparison of *Medium-High* and *High-High* workflows in top-down analysis of Ec70S ribosomes. a) Top-down LC-MS/MS workflows with medium resolution MS1 and high resolution MS2 (*Medium-High* analysis) provide better coverage of the ribosomal proteome. Short transient scans, although they have lower resolution, provide better signal to noise ratio than long transient, high resolution scans. b) This allows larger proteins to be selected for fragmentation more frequently, covering the entire *E. coli* 70S ribosome proteome, as can be seen in a typical base peak chromatogram.

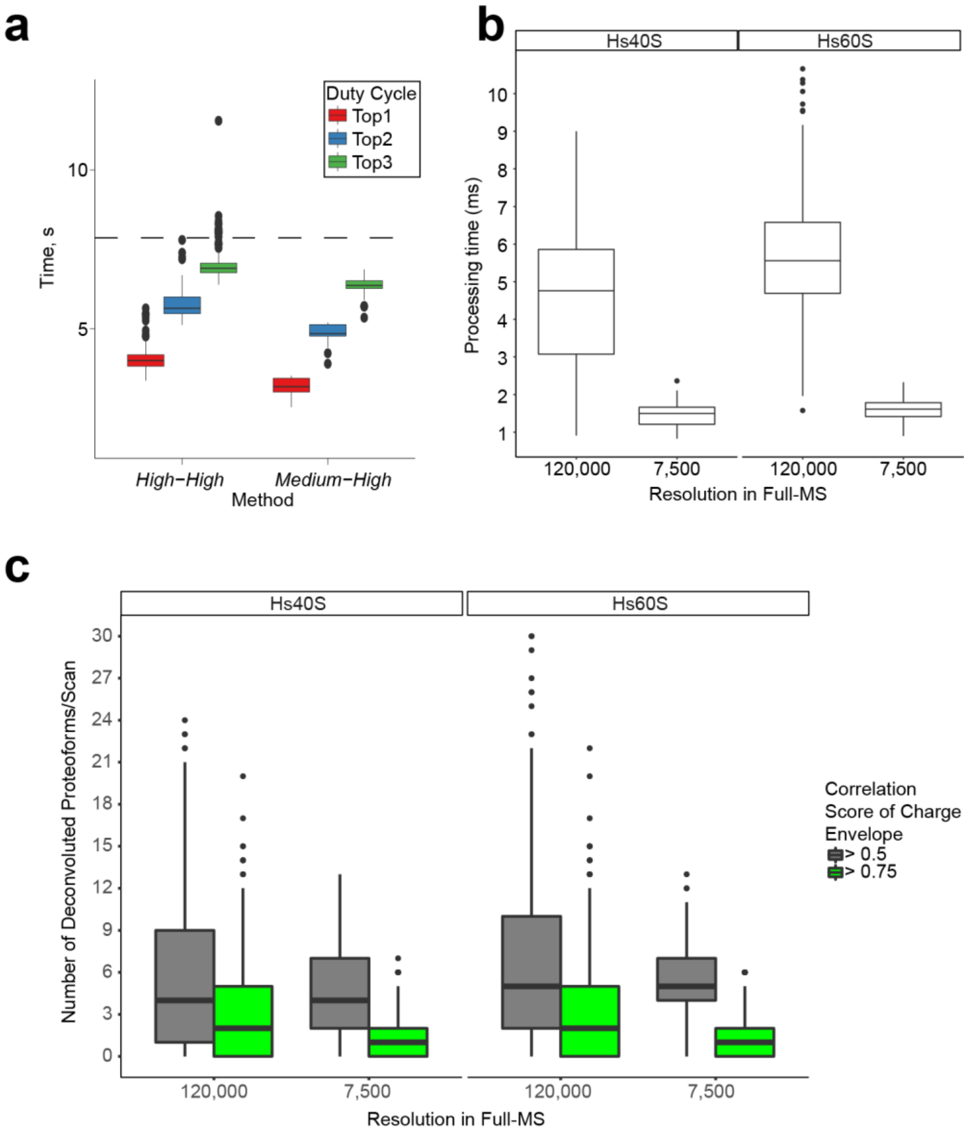

Supplementary Figure 3. Performance comparison of *Medium-High* and *High-High* workflows in top-down analysis of Hs40S and Hs60S ribosomes. a) Duty cycle times for TopN (N = 1, 2, or 3) *High-High* and *Medium-High* methods compared to the shortest elution window observed for human ribosomal proteins. The data indicate that the *Medium-High* method provides means for faster sequencing of proteoforms in samples of higher complexity. b) Processing time of on-the-fly deconvolution algorithms for isotopically-resolved full MS spectra (recorded at 120,000 resolution setting) and unresolved spectra (recorded at 7,500 resolution setting) for human ribosomal particles. The processing time of the algorithm for high-resolution spectra increases with increase of sample complexity, while the deconvolution algorithm for unresolved spectra proves to be faster and shows less dependence on sample complexity. c) The detected number of deconvoluted proteoforms per scan demonstrates that high-resolution full MS is more sensitive to picking more reliable proteoforms. The correlation score reflects how well the observed charge envelope resembles the predicted charge envelope.

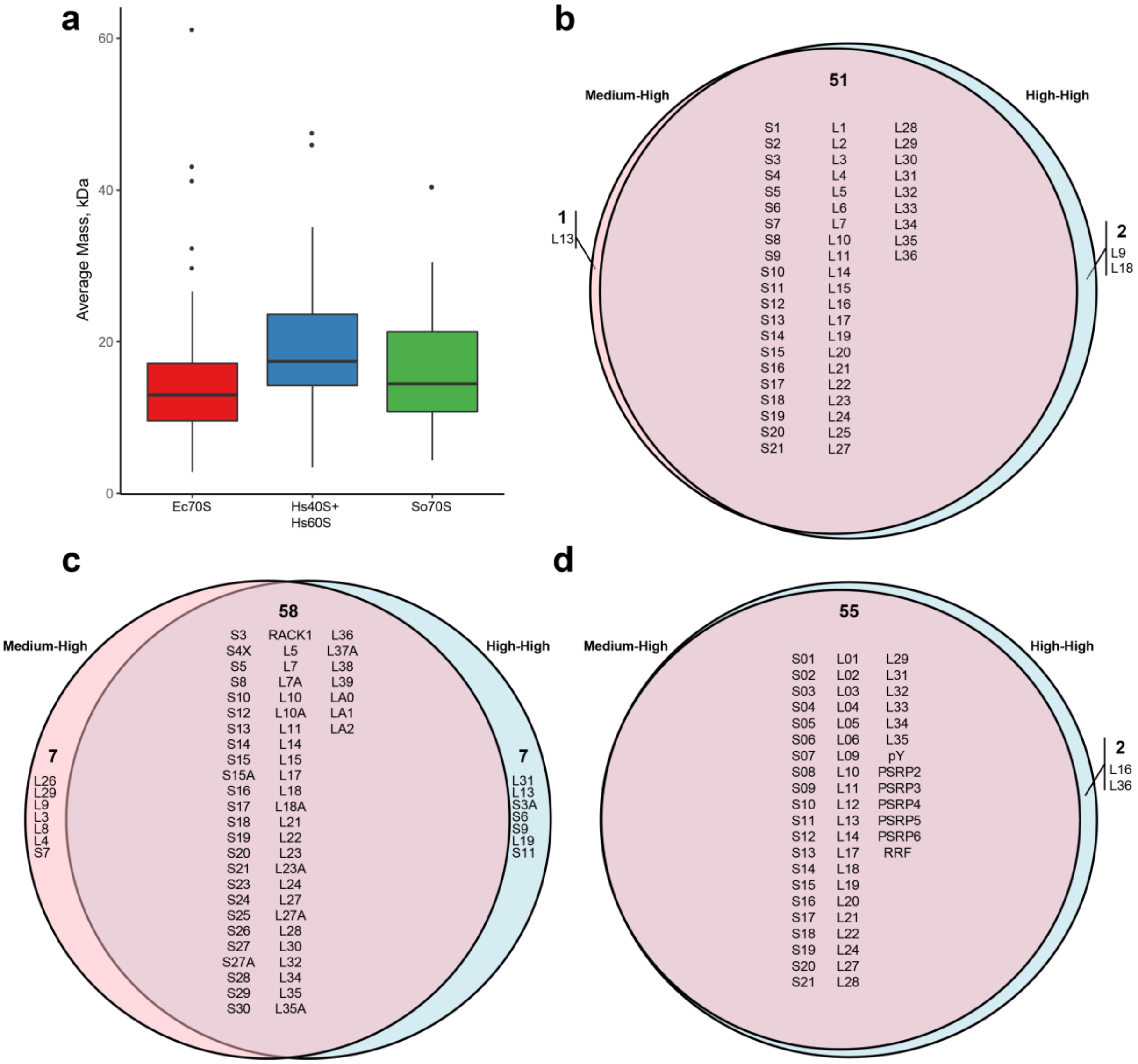

Supplementary Figure 4. Comparison of detected proteins by *Medium-High* and *High-High* workflows in top-down analysis. a) Distribution of the theoretical average masses of the proteins in the analyzed ribosomal particles. b-d) Venn diagrams showing the ribosomal proteins identified by database searching with Prosight PD by using either *High-High* (blue) or *Medium-High* workflows (pink). The results are shown for the distinct ribosomal particles: b) Ec70S, c) Hs40S and Hs60S, and d) So70S.

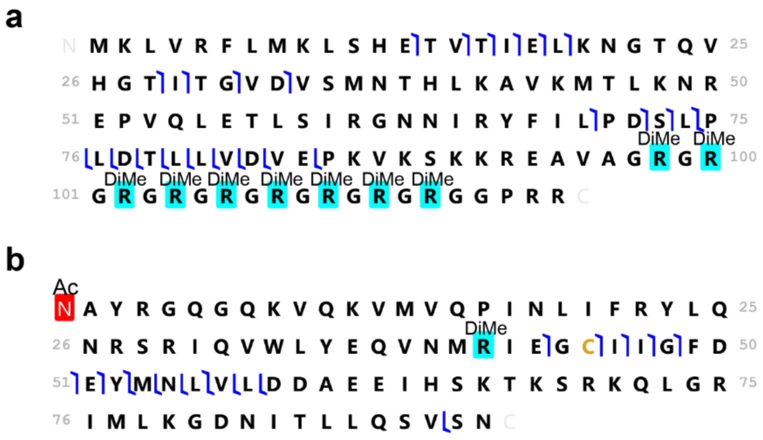

Supplementary Figure 5. Fragmentation maps of two human spliceosome proteins co-purified in the human ribosomal purification. a) Small ribonucleoprotein D1 with manually assigned 9 arginine dimethylations, localized in the Gly-Arg rich C-terminus. b) Small ribonucleoprotein E with removed methionine and unreported dimethylation. For both proteins, unambiguous determination of the exact site locations of the post-translational modifications requires more extensive analysis with a higher number of fragments.

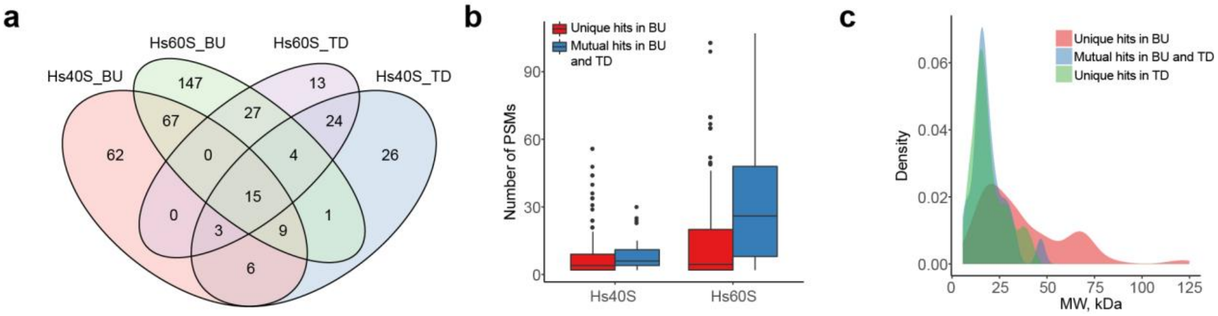

Supplementary Figure 6. Comparison of the protein identifications in the top-down and bottom-up analyses of the 40S and 60S human ribosomal samples. a) Venn diagram of protein identifications in the bottom-up (BU) and top-down (TD) analysis of the Hs40S and Hs60S purified particles. b) Proteins identified in the top-down analysis comprise only a fraction of those identified in the bottom-up analysis, mostly those high number of peptide spectrum matches (PSMs), while proteins identified with fewer PSMs were detected solely in the bottom-up analysis. c) Density plot for the mass distribution of the proteins mutually or uniquely identified in the bottom-up and top-down LC-MS/MS analyses. In all these comparisons, results form a single bottom-up LC-MS/MS analysis was compared with merged results from 3-6 top-down LC-MS/MS analyses. Only proteins with at least two unique peptides identified and high confidence of identification were taken into account in this comparison.

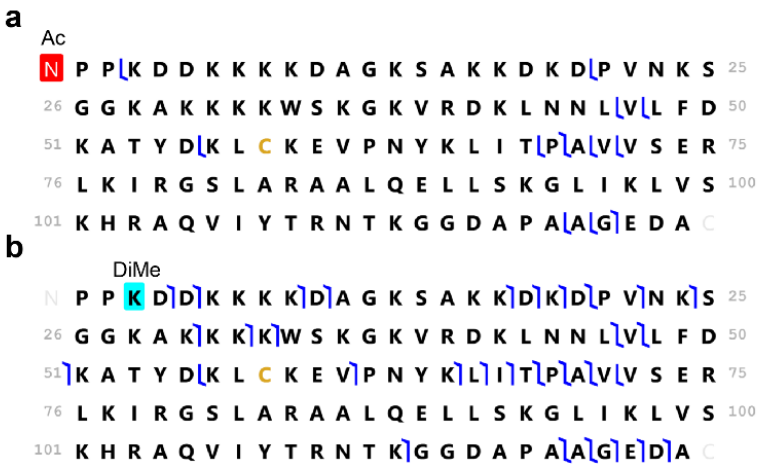

Supplementary Figure 7. Manual annotation reveals Human S25 to be dimethylated at Lys-3. a) Incorrectly assigned proteoform of the human S25 as N-terminally acetylated in Prosight PD *versus* b) correct positioning of an unreported dimethylation at Lysine 3. Upon manual annotation the P-score for identification of S25 protein improved from 2e-11 to 1.1e-52 for the most intense protein spectrum match.

a **b**

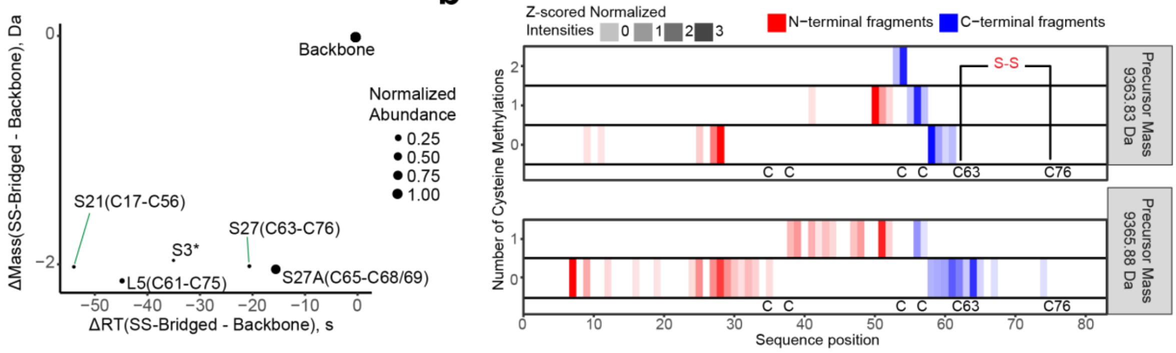

c

	Observed	Theoretical	Error, ppm	Composition
3CysMet	9365.8762	9365.8422	3.64	C411H671N121O115S7
3CysMet+S-S Bridge	9363.8493	9363.8266	2.44	C411H669N121O115S7

Supplementary Figure 8. Top-down analysis reveals co-occurrence of reduced and non-reduced disulfide bridges in human ribosomal proteins. a) Disulfide linked proteoforms can be distinguished from their reduced forms by shifts in retention time and intact mass in Top-down LC-MS/MS as is shown for 5 human ribosomal proteins (S27, S3, S21, S27A and L5). The dot size displays abundance relative to abundance of the primary proteoform. b) Fragmentation of LC-separated disulfide-bridged and primary proteoforms of S27 human ribosomal protein. c) Accuracy of deconvoluted precursor masses of S27 with and without disulfides further supports the unambiguous identification and characterization.

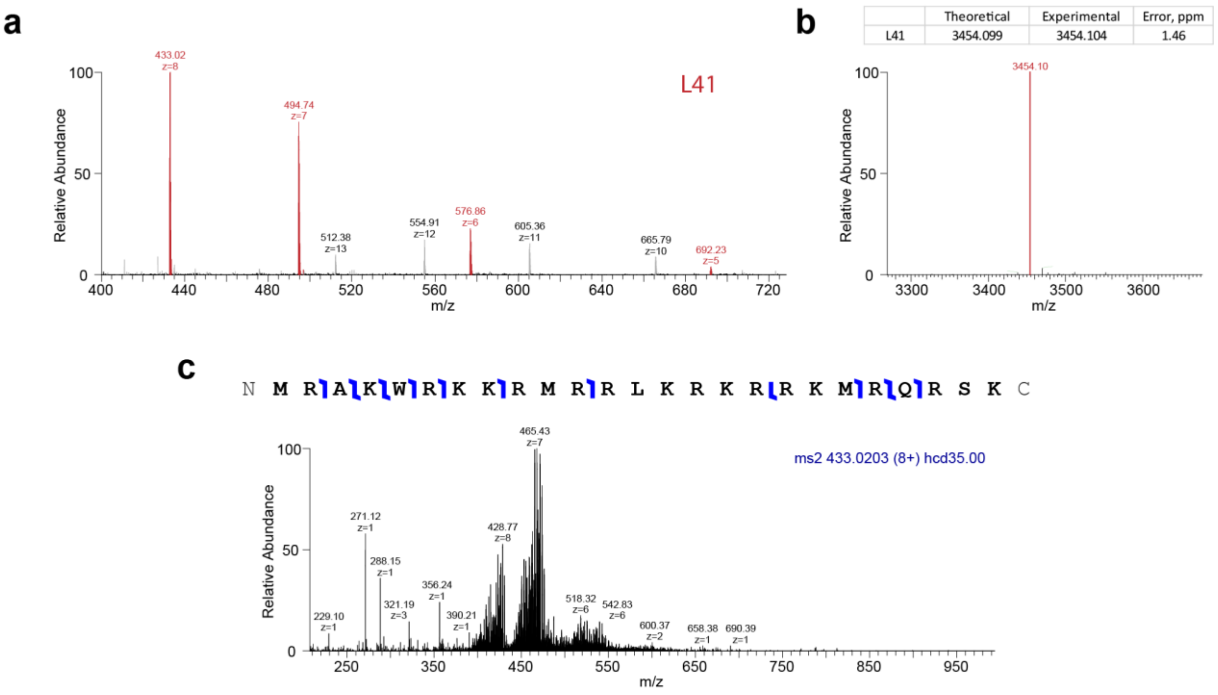

Supplementary Figure 9. Confirmation of the presence of the L41 ribosomal protein in the Hs40S ribosomal purifications by top-down LC-MS/MS. a) Full MS spectrum of the human ribosomal protein L41. b) The extracted deconvoluted mass of L41. c) Fragmentation map and HCD MS2 spectrum of isolated z = 8+ ions of L41.

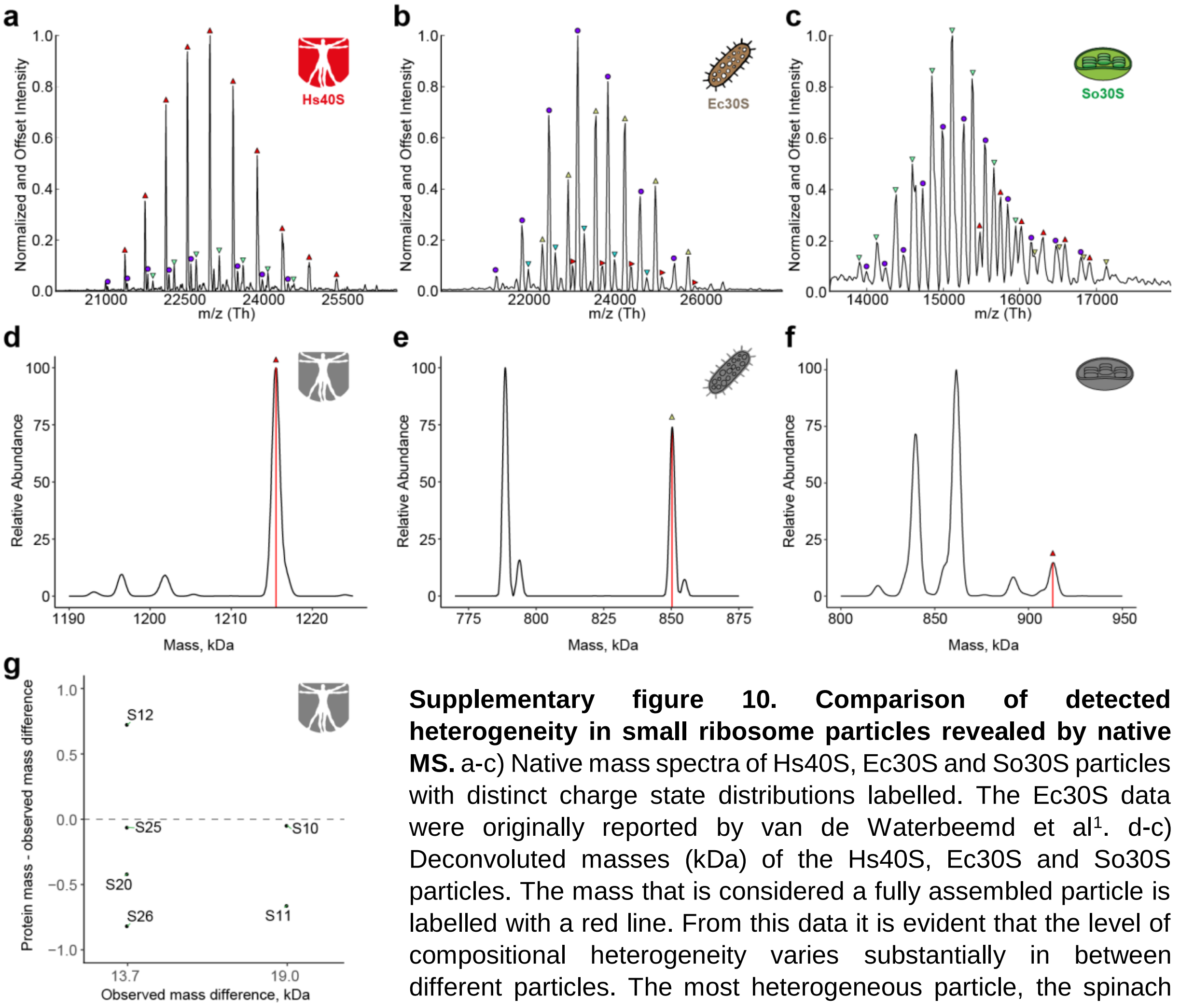

Supplementary figure 10. Comparison of detected heterogeneity in small ribosome particles revealed by native MS. a-c) Native mass spectra of Hs40S, Ec30S and So30S particles with distinct charge state distributions labelled. The Ec30S data were originally reported by van de Waterbeemd et al[1]. d-c) Deconvoluted masses (kDa) of the Hs40S, Ec30S and So30S particles. The mass that is considered a fully assembled particle is labelled with a red line. From this data it is evident that the level of compositional heterogeneity varies substantially in between different particles. The most heterogeneous particle, the spinach So30S, requires further investigation to fully explain the sources of heterogeneity. g) The 13.7 and 19 kilo-Dalton mass differences in the Hs40S particles can be confidently linked to the loss of the proteins S25 and S10, respectively. The pictograms of the Vitruvian man were prepared based on an image that was obtained under a CC0 license from https://commons.wikimedia.org/wiki/File:Digisapiens.png.

a

	Full Proteome		Shuffled Full Proteome	
	Protein Groups	PSMs	Protein Groups	PSMs
Ec70S	56	989/1054	0	0/0
Hs40S	135	1749/2384	0	0/0
Hs60S	98	1409/1660	2	1/4
So70S	65	2497/2615	2	2/2

b

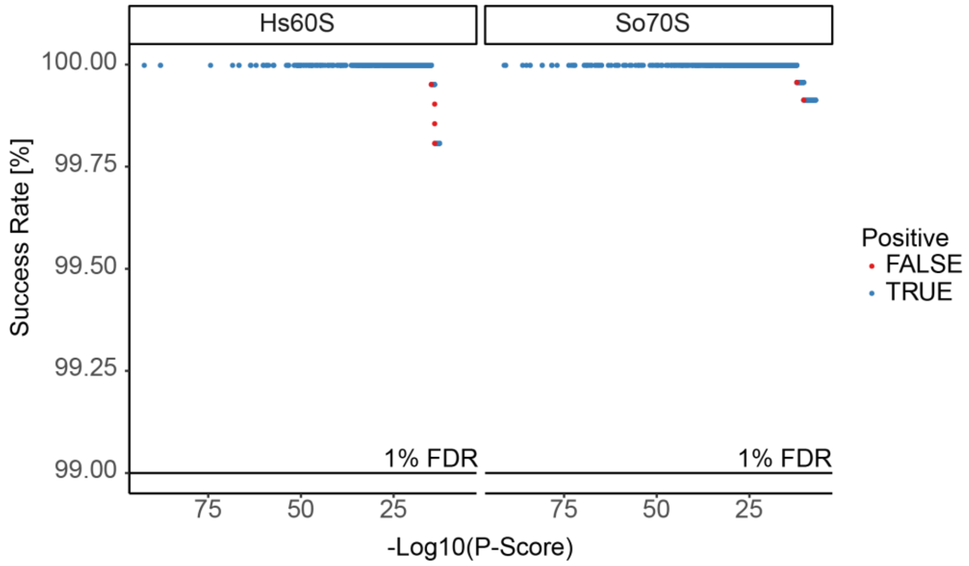

Supplementary Figure 11. Estimated false discovery rates in top-down database searches. a) Identification statistics for database searches of top-down LC-MS/MS of Ec70S, Hs40S-Hs60S, and So70S ribosomal particles against full proteomes of *E. coli* (strain K12), *H. sapiens*, and *S. oleracea*, respectively. PSMs are represented as follows: Unambiguous PSMs/All PSMs. b) Cumulative error based on false positive protein spectrum match calculated against decreasing $-\log_{10}$(P-Score) for Hs60S and So70S ribosomal particles, for which false positive hits against shuffled databases were observed. This estimates the false discovery rate to be around 0.25%, well below the generally accepted 1%.

1. van de Waterbeemd, M. *et al.* High-fidelity mass analysis unveils heterogeneity in intact ribosomal particles. *Nat. Methods* **14,** 283–286 (2017).
