# Supplementary material for: Dissecting ribosomal particles throughout the kingdoms of life using advanced hybrid mass spectrometry methods
Source: Nat Commun. 2018 Jun 27;9:2493. doi: 10.1038/s41467-018-04853-x (PMC6021402; doi:10.1038/s41467-018-04853-x)
Supplement: Supplementary file 2 — Description of Additional Supplementary Files [file 41467_2018_4853_MOESM2_ESM.pdf]

Descriptions of Additional Supplementary Files

File Name: Supplementary Data 1
Descriptions: Proteins identified in the bottom-up LC-MS/MS analysis of Hs40S, Hs60S, and So70S ribosomal purifications. The ranking of proteins is performed following the iBAQ values for each ribosomal protein in the purifications.

File Name: Supplementary Data 2
Descriptions: Ribosomal proteins identified in top-down LC-MS/MS of So70S chloroplactic ribosomal particle. Sequences available in databases were manually adjusted with sequence processing events confirmed in protein spectrum matches.

File Name: Supplementary Data 3
Descriptions: Theoretical average masses of proteins and experimental average masses of the most abundant proteoforms of 40S human ribosomal subunit with corresponding relative abundances.
